# Supplementary material for: Surgical Outcomes and Trends for Chronic Pancreatitis: An Observational Cohort Study from a High-Volume Centre
Source: J Clin Med. 2022 Apr 9;11(8):2105. doi: 10.3390/jcm11082105 (PMC9027315; doi:10.3390/jcm11082105)
Supplement: Supplementary file 1 [file jcm-11-02105-s001.zip › jcm-1625270-supplementary.pdf]

Table S1. Non-surgical data before and after surgery for the entire cohort.

	Prior to surgery<br>*n* = 162	6 months after surgery<br>*n*=162	p value[a,b,c]	Missing cases (*n*)
PEI, *n* (%)	70 (45)	142 (90)	**< 0.001**	5
PERT dosage in lipase units, median (IQR)	0 (0 – 150 000)	150 000 (84 000 – 225 000)	**< 0.001**	26
BMI in kg/m² median (IQR) and mean (± SD)	23.5 (20.9 – 28.1)	22.7 (19.4 – 25.7)	**< 0.001**	48
Stool consistency, *n* (%)			0.889	44
Constipation	6 (5)	7 (6)		
Normal	86 (73)	85 (72)		
Diarrhoea	26 (22)	26 (22)		
DM, *n* (%)	48 (31)	77 (50)	**< 0.001**	9
Pain treatment, *n* (%)				
Acetaminophen	60 (44)	61 (45)	1.000	26
NSAID	17 (12)	9 (7)	0.115	26
Opioid	54 (40)	42 (31)	0.058	26
Opioid dosage in mg, median (IQR)	0 (0 – 36)	0 (0 – 10)	0.060	34
Pain intensity, *n* (%)			**< 0.001**	41
No pain	52 (43)	79 (65)		
Mild/moderate pain	38 (31)	30 (25)		
Severe pain	31 (26)	12 (10)		

[a] Comparison between prior to surgery and 6 months after surgery for entire cohort.
[b] p values were calculated using McNemar´s test for categorical variables and Wilcoxon signed – rank test for non-normally distributed variables and ordinal data.
[c] Bold values denote statistical significance ($p < 0.05$).
PEI: Pancreatic exocrine insufficiency; PERT: Pancreatic enzyme replacement therapy; IQR: Interquartile range; BMI: Body mass index; DM: Diabetes Mellitus; NSAID: Non-steroidal anti-inflammatory drug; DPPHR: Duodenum-preserving pancreatic head resection; PD: Pancreaticoduodenectomy.
